# Value-Based Healthcare in Ostomies

**DOI:** 10.3390/ijerph17165879

**Published:** 2020-08-13

**Authors:** Ana C. Montesinos Gálvez, Francisco Jódar Sánchez, Carmen Alcántara Moreno, Antonio J. Pérez Fernández, Rosario Benítez García, Mercedes Coca López, María Paz Bienvenido Ramírez, Monserrat Cabrera López, Luisa Vázquez Burrero, Pilar Jurado Berja, Raquel Sánchez García, Josefa Martín Cebrián, María Luz Hervas García, Remedios López Fernández, Claudia Pérez Jiménez, María Antonia Reyes Vico, Ana Belén Vargas Villegas, Nuria García-Agua Soler, Antonio J. García Ruiz

**Affiliations:** 1 Hospital Regional Universitario de Málaga, 29010-Málaga, Spain; acmontesinos@uma.es; 2 Faculty of Economics, University of Málaga, 29001-Málaga, Spain; fjodar@uma.es; 3 Hospital Universitario “Virgen de la Victoria”, 29010-Málaga, Spain; carmenalmo79@hotmail.es; 4 Hospital de St Ana. Motril, 18600-Granada, Spain; antonioj.perez.fernandez.sspa@juntadeandalucia.es; 5 Hospital Universitario de Puerto Real, 11510-Cádiz, Spain; charibenitezgarcia@gmail.com (R.B.G.); relopfe@gmail.com (R.L.F.); 6 Hospital Universitario “Nuestra Señora de Valme”, 41014-Sevilla, Spain; merchicoca@hotmail.com; 7 Hospital Universitario Puerta del Mar, 11009-Cádiz, Spain; mpbrmaripi@gmail.com; 8 Hospital de Poniente, El Ejido, 04700-Almería, Spain; montcl6@hotmail.com; 9 Hospital “Juan Ramón Jiménez”, 21006 Huelva, Spain; burreroluisa@gmail.com; 10 Complejo Hospitalario de Jaén, 23007-Jaén, Spain; pjuradoberja@yahoo.es; 11 Hospital de Pozoblanco, 14400-Córdoba, Spain; raquel.sanchez.garcia.sspa@juntadeandalucia.es; 12 Hospital Universitario Virgen de la Nieves. 18014-Granada, Spain; carmencastellano_i64@hotmail.com; 13 Hospital San Agustin (Linares). 23700-Jaén, Spain; mayrahervas@gmail.com; 14 Hospital Costa del Sol, Marbella, 29603-Málaga, Spain; pjclau@gmail.com; 15 Hospital San Juan de la Cruz (Übeda), 23400-Jaén, Spain; reyesvico@hotmail.com; 16 Hospital General de Riotinto, 21660-Huelva, Spain; anab.vargas.sspa@juntadeandalucia.es; 17 Department of Pharmacology, School of Medicine, University of Málaga, 29010-Málaga, Spain; nuriags@uma.es; 18 School of Medicine, University of Málaga, 29010-Málaga, Spain

**Keywords:** ostomy, organizational innovation, nurse specialists, quality-adjusted life years, health care costs, cost-benefit analysis, health resources, prospective studies

## Abstract

In order to achieve significant improvements in quality, cost, and accessibility (the health “iron triangle”), innovation in organizational and service delivery models is necessary to increase the value of healthcare. The aim of this study is to evaluate the efficiency of a model of organizational innovation based on advanced practice nurse in the care of people with ostomies (APN-O) versus usual care. An observational, exploratory, analytical, prospective study with a six-month follow-up was carried out at 12 hospitals that implemented this model in Andalusia. A total of 75 patients who had undergone a digestive elimination ostomy and/or a urinary ostomy were followed for six months. Clinical outcomes, healthcare resources, health-related quality of life, and willingness to pay (WTP) were analyzed. The economic evaluation was conducted from a societal perspective, including healthcare costs and indirect costs. The cost difference between the two models was €136.99 and the quality-adjusted life year (QALY) gained was 0.05965 (€2297 per QALY gained). At six months, the mean of WTP was €69 per APN-O consultation. This model contributes to increasing the value-based healthcare in ostomies. Results of this study suggested that APN-O is an effective patient management model for improving their health status and is highly efficient.

## 1. Introduction

An ostomy, a surgical procedure that connects a section of the intestine to the surface of the body via the abdominal wall, is a therapeutic solution for certain intestinal problems [1,2]. The main reason for this type of procedure is colorectal cancer, which makes up 80% of all ostomies performed in Spain [3]. Although no official data exists, it is estimated that 1.5 out of every 1000 Spanish citizens has an ostomy. This equates to 70,000 people, with over 13,000 new cases every year [4,5]. In other Western countries, figures vary between 2 and 4 out of every 1000 adults [5]. Globally speaking, colorectal cancer ranks twenty-third in terms of burden of disease [6], accounting for the loss of more than 17 million disability-adjusted life years (DALYs).

In order to achieve significant improvements in quality, cost, and accessibility (the sanitary “iron triangle”), we need to implement innovative organizational and service provision models with the aim of increasing the value of healthcare (value-based healthcare). This is a new sanitary paradigm, born from technological advancements, scientific innovation, and changes in patient health culture. It involves a break away from traditional medicine, based on the quantity of services provided, to one based on the value of these services. That is to say, a quality-based service. To this effect, and in order to achieve improved healthcare results, it is necessary to know the relation between results that are relevant for the patient and costs per patient throughout the complete cycle of care [7,8].

The roles of advanced nursing, which arose to respond to the needs of the population, go beyond the traditional boundaries of the nursing profession towards a competitive evolution that is today known as advanced practice nursing (APN). The International Council of Nurses (ICN) defined APN as a registered nurse who has acquired the expert knowledge base, complex decision-making skills, and clinical competencies for expanded practice. The specifics of such are shaped by the context and/or country in which the nurse is certified to practice. They must be recognized as highly qualified health providers, have specific experience, and provide added value to the health system within which they are developing their competencies [9].

The role of stoma care nurses, annual follow-ups, and initial and long-term education has developed, and is essential to prevent poor use of the devices and accessories. This can lead to reduced prescription costs, have a positive effect on patients’ health-related quality of life (HRQL), and reduce issues associated with adverse events, healthcare resources, and healthcare costs [10,11,12,13]. Although there are several studies on the improvement in quality of life of ostomy patients, such as those cited above, none of them evaluate a new model of comprehensive patient management such as the one that has been implemented in our region.

In our region, Andalusia, within the framework of comprehensive health plans and integrated care processes [14], nursing competencies and specific profiles have been developed to provide high quality care in a safe environment that allows for prevention, health promotion, and patient recovery, thus improving overall quality of life [15]. The competential development framework of nurses in the Andalusian Public Health System has been defined according to the APN competency assessment instrument [16] and the advanced practice nurses competences and functions defined by Miguélez-Chamorro et al. [17]. APN core competencies to develop their role in the Andalusian Public Health System are [18]: research and evidence-based practice, clinical and professional leadership, interprofessional relations and mentoring, quality management and care management, and professional teaching and education. Therefore, a model of organizational innovation based on APN in the care of people with ostomies (APN-O) should contribute to increasing the value of health care (Value-Based Health Care), thus improving the patient’s HRQL and reducing costs associated with the consumption of healthcare resources and productivity losses.

The aim of this study is to evaluate the efficiency of an organizational innovation model based on APN-O versus usual care, something that until now and despite numerous particular studies mentioned above, does not evaluate the patient’s integral process, that is, the new management model.

This study should serve to provide information to help clinical managers determine whether implementing this innovation generates more value in health care (based on effectiveness and efficiency criteria).

## 2. Materials and Methods

### 2.1. Study Design and Scope

Observational, exploratory, analytical, prospective study with a six-month follow-up. The study population was surgical patients who had undergone an elimination digestive and/or urinary ostomy and who met the following inclusion criteria: having undergone abdominal surgery with an elimination digestive and/or urinary ostomy; no cognitive deficit; voluntary access to the study through informed consent. Exclusion criteria were: people with feeding ostomies (i.e., gastrostomized) or respiratory ostomies; patients who presented a language barrier. Inclusion of patients was carried out between November 2018 and July 2019.

The scope of the study corresponded to 12 hospitals that had implemented the APN-O management model in Andalusia (autonomous community of Spain): one regional hospital, six specialized hospitals, and four county hospitals.

### 2.2. Study Variables

The variables analyzed for each patient were as follows:

- Sociodemographic: age, gender, civil status, household members, education level, household economic level.
- Clinical: illness that caused the ostomy, type of surgical intervention, type of ostomy, stoma complications, and/or postoperative treatments.
- Healthcare resources: these were estimated based on the consumption of health resources associated with ostomy care, follow-up, revision, and complications: number of visits to hospital emergency departments and number of hospitalizations, number of visits to specialised nurses (APN-O), visits to specialized care (medical specialists), number of visits to primary care (doctor and/or nurse) and primary care emergency services.
- Health-related quality of life. Two surveys were used:
  - Stoma Quality of Life Index-Montreux [19], an ostomy-specific survey. It includes 41 questions that collect information on the quality of life (physical well-being, psychological well-being, body image, pain, sexual activity, nutrition, social worries, and handling of devices), self-sufficiency (related to the hygienic care of the stoma) and general issues (related to the acceptance of the stoma and family relations). The score obtained ranges from 0 (worse quality of life) to 100 (better quality of life).
  - The generic EuroQol-5D-5L survey [20], which describes the state of health across five areas (mobility, personal care, daily activities, pain/discomfort, and anxiety/depression). It also includes a graph, the visual analog scale (VAS), on which the subject must rate the state of their health on a scale of worst imaginable state of health (0) to the best imaginable state of health (100). Health state utility values can be obtained from this survey.
- Willingness to pay: the willingness to pay (WTP) was evaluated through a contingent valuation method. To this effect, we described a hypothetical scenario to the patient in which they had to establish their WTP for complete and comprehensive care starting from the stomal therapy consultation, using their WTP for each APN-O consultation.

### 2.3. Sample Size

The calculation of the sample size was made taking into account the proportion of patients who improve their health status from the beginning to the end of the study, according to the EuroQol-5D-5L questionnaire, and report no health problems. Thus, for a unilateral test with a 95% confidence level, a statistical power of 80%, a proportion at the beginning of the study of 40% and 65% at the end of the study (improvement of more than 20% of patients in HRQL) and adjusting for 15% losses, the sample size was 71 patients. The calculation of the sample size was done by using the GRANMO online calculator [21,22].

### 2.4. Model of Organizational Innovation Based on APN-O

Innovative organizational model identifies the following APN-O interventions [18]:

- Interventions of diagnostic stage: (i) accompany patients through preferential circuits providing personalized care and specific information; (ii) manage and expedite diagnostic tests prior to the treatment option; (iii) inform and advise on an individual basis on the steps of the process; (iv) help to improve individual and family coping.
- Interventions of presurgical stage: (i) marking and signalling the best area to perform the outcrop of the ostomy, which involves fewer problems and periostomal complications.
- Interventions of therapeutic stage: (i) planning of care according to the needs detected after the comprehensive assessment and with the resources available in each centre; (ii) coordination of resources between the different services in the hospital environment and between the different care areas that favours the coordination of the therapeutic plan to ensure continuity of care and accessibility to the services and professionals involved in the processes; iii) proactive monitoring with the intention of detecting signs and symptoms early, resolving doubts, and mitigating fear; iv) emotional support: enhancing security, self-esteem, coping with the situation
- Interventions of discharge of the service: (i) transfer the information to the following assistance service; (ii) guarantee support systems and continuity of care.

### 2.5. Data Collection

The collection of clinical data, HRQL and WTP was carried out prospectively. Variables associated with the consumption of healthcare resources were collected from medical records. Patients were recruited in phases until the proposed sample size was reached.

- Recruitment visit (baseline visit): data collection related to sociodemographic and clinical variables, HRQL and WTP.
- Final visit (six months): data collection related to HRQL, consumption of healthcare resources and WTP.

The visits for the study were made to coincide, whenever possible, with those established in the follow-up criteria of the respective center’s care assistance processes, thus preventing the patient from repeatedly visiting the hospital.

### 2.6. Economic Evaluation of the New Management Model Based on APN-O

The economic evaluation was carried out following the recommendations of clinical and economic evaluation guides applied to national [23] and international health technologies [24,25]. The economic evaluation was conducted from a societal perspective, including healthcare costs and indirect costs.

Costs: the costs of healthcare resources were estimated using the price list published by the Andalusian Public Health System [26,27] and other related publications [28]. Indirect costs related to the productivity loss were estimated based on labor costs. The results were shown in euros of the base year, updated according to the Consumer Price Index. Lost labor output associated with medical leave, from the surgery date until the patient’s re-entry into normal activity, was included in the calculation of productivity loss. Labor cost is defined as the cost incurred by the employer for their use of the work factor. This term measures the cost posed to the employer by employing a worker during one month. It was estimated using the Quarterly Labor Cost Survey [29]. All analyzed costs are shown in Table 1.

Cost–utility analysis: a cost–utility analysis was carried out to compare effectiveness and cost outcomes of the model of organizational innovation based on APN-O (postintervention period) versus usual care (preintervention period without APN-O). For the preintervention period, we assumed the same healthcare resource consumption of the six months of follow-up except for those derived from the new model. In addition, we assumed the baseline level of utility, assuming that the patient’s quality of life remained constant throughout the six months preintervention. We calculated the utility gained (effectiveness) for each patient in terms of quality-adjusted life years (QALYs) since the start of the study. That is to say, the gradual effectiveness. The final results were expressed in terms of the incremental cost-effectiveness ratio. The QALY was estimated from the score of the EuroQol-5D-5L, which gave a health profile and a single index on HRQL [30].

Cost–benefit analysis: This analysis compares the costs and the savings associated with APN-O consultations. It also incorporates patients’ health benefits in monetary in terms of WTP.

### 2.7. Statistical Analysis

For the quantitative variables, we obtained: mean, standard deviation, and the 95% confidence interval for the mean. For the qualitative variables we obtained frequency and proportion. The following analyses were used for the comparison between 2 variables in inferential statistics:

- Student’s *t*-test or the Mann–Whitney U test (nonparametric statistics) were used when comparing two quantitative variables.
- the chi-squared test (with Yates’s or Fisher’s correction, as applicable) when comparing two qualitative variables.
- One-way analysis of variance (ANOVA) or Kruskal–Wallis test (nonparametric statistics) were used to determine whether there were any statistically significant differences between the means for more than two groups).
- Wilcoxon signed-rank test were used to compare two related samples.

The SPSS v24 software, licensed for use by the University of Málaga, was used for the statistical analysis.

### 2.8. The Effect Size

A statistically significant difference is not necessarily a large difference, nor is it necessarily an important (relevant) difference. Thus, a statistically significant value of a test will allow us to confirm that the difference between means of populations represented by those two samples is not zero (more precisely, that it is very unlikely to be zero). However, there is no relation between the value of the test and the magnitude of the difference. In order to differentiate between statistically significant and clinically relevant results, calculations corresponding to the effect size will be performed [31,32]. Through the index known as Cohen’s d, we can determine how strong the association between two variables is, or what differences they have. We used an Excel page with open-source software to perform the calculations from the means and their standard deviation or standard error [33]. Cohen’s d index allows us to quantify the effect of the treatments in relation to the analyzed clinical criteria. This can be an: insignificant effect (−0.15 and <0.15); small effect (≥0.15 and <0.40); medium effect (≥0.40 and <0.75); large effect (≥0.75 and <1.10); very large effect (≥1.10 y and 1.45); enormous effect >1.45 [34,35].

### 2.9. Ethical Considerations

This research project did not foresee or implement any intervention in terms of the patient’s treatment, nor did it extract biological samples.

The research team pledged to follow current legislation regarding biomedical research, specifically the Declaration of Helsinki (current version, Fortaleza, 2013), Organic Law 15/1999 on the Protection of Personal Data and the European Parliament and Council Regulation (EU) 2016/679 of 27 April 2016 on the protection of natural persons with regard to the processing of personal data and on the free movement of such data, and repealing Directive 95/46/EC (General Data Protection Regulation). The access to clinical data was restricted to research staff, who are also the healthcare personnel taking care of the patients.

According to Spanish Law 14/2007 of July 3rd on biomedical research, all subjects gave their informed consent for inclusion before they participated in the study. The protocol was approved by the Ethics Committee of Málaga (1-10-2018) PEIBA code: 1420-N-18.

The recruiting researcher, from each of the hospitals taking part, explained to each patient (or legally authorized representative) the nature of the study, its aim, procedures, expected duration, and potential risks and benefits regarding the participation in the study, as well as any inconvenience this might cause. The informed consent was provided in writing, in an easily understandable language for the participant. No patient was included in the study without having provided their informed consent.

## 3. Results

### 3.1. Sociodemographic and Clinical Analyses

A total of 75 patients were recruited for the study, all of which were examined according to the study protocol. The sociodemographic basal and clinical characteristics of patients included in the study are shown in Table 2 and Table 3. The mean age was 61 years (range: 25–90 years). Most were men (56%), married or living with a partner (70.7%), with primary education (35.3%) and employees (53.3%).

The most frequent reason for surgery was oncological (74.7%), and the most frequent intervention was elective surgery (59.5%). The most frequent ostomy type was colostomy (59.4%) and the majority of them did not require postoperative treatment (38.8%). Only 5.4% of patients did not receive the preoperative marking in case of proceeding (elective surgery).

There were no statistically significant differences between the type of hospital and the sociodemographic variables. There were no differences regarding clinical characteristics either, except for the preoperative marking, where there were differences between regional hospitals (more marking performed) and the other types of hospitals (*p* = 0.024).

### 3.2. Use of Healtcare Resources Due to Complications with the Stoma

About one third of patients that completed the study (37.9%) did not have any complications with their stoma. One third of them (33.3%) presented surgical complications, the most frequents being shrinkage (13.6%) and parastomal hernia (10.6%), and 28.8% of patients had complications arising from the stoma, with dermatitis (21.2%) and mucosal edema (4.5%) among the most frequent. There were statistically significant differences between the number of patients with complications and type of hospital (Table 4).

Ninety-one percent of patients of patients (n = 60) did not need to go to the hospital emergency services even once when they experienced complications with their stoma; from the rest, 7.6% attended once (n = 5) and one patient attended four times. Only 3.1% had to be admitted in the hospital due to complications with the stoma, two patients once and one patient twice. The mean number of days patients were admitted for was 0.06 (SD: 0.39 days).

In terms of the type of professional involved in the resolution of stoma problems and surgical complications, 30.8% of patients visited the hospital specialist (surgery), the mean of visits by patient being 0.65 (SD: 1.20); 7.6% attended their primary health care doctor (0.32; SD: 1.47); 12.1% were given primary healthcare nursing (0.59; SD: 2.75) and 7.6% attended healthcare emergency services (0.12 SD: 0.44).

Data correspondent to visits to specialized nursing are shown in Table 5. Only in terms of the type of visit and emergency demand between county and specialized hospitals were there any differences. (*p* = 0.034).

### 3.3. Health-Related Quality of Life

**Figure 1:**
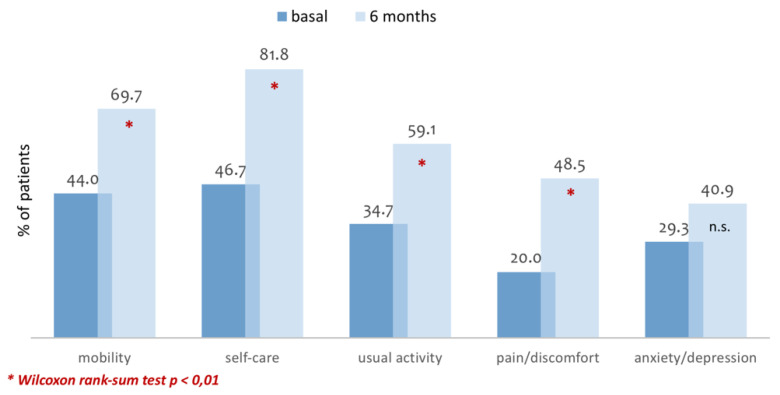
Percentage of patients that express not having any health problems (EQ5D5L).

When it comes to quality of life measured with the generic questionnaire EuroQol 5D5L, statistically significant differences were found between the beginning and the six months of study (Figure 1) in all dimensions, except for “anxiety/depression”. With regards to the health condition of the patient, measured with the visual analogic scale (0 being worst health condition and 100 being best health condition), it went from 58.4 at the beginning of the study to 73.03 at the end (*p* = 0.0001; Cohen’s d = 0.68).

The utility calculated at the beginning of the study was 0.5807 (SD: 0.3295), whereas at the end of the study it was 0.8193 (SD: 0.1677). These differences were statistically significant (*p* < 0.001) and clinically relevant (Cohen’s d = 0.91). The QALY gained after six months was 0.05965 (SD: 0.0731). This gain in utility was statistically significant (*p* < 0.001) and clinically relevant, Cohen´s d = 0.7579), which corresponds to a size of large effect.

**Figure 2:**
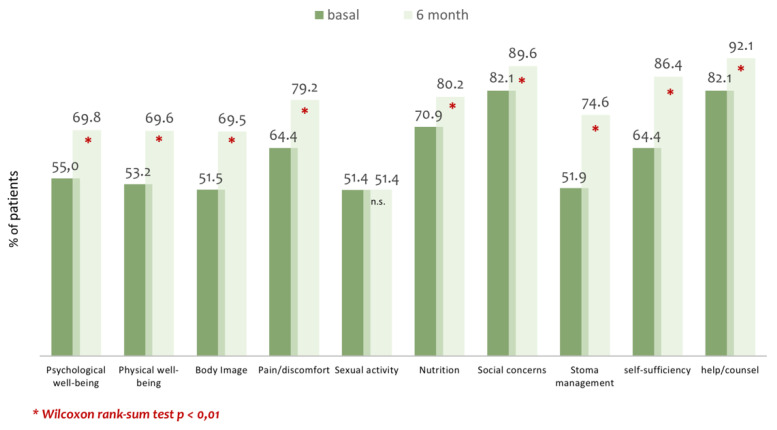
Mean score obtained with the Stoma Quality of Life Index specific survey. Range 0 = worse quality of life; 100 = better quality of life.

The quality of life measured with the Stoma Quality of Life Index between the beginning and the six-months of study improved gradually across all areas, and these differences were statistically significant (*p* < 0.01) except for dimensions concerning sexual activity and help. Results for each section of the questionnaire are shown in Figure 2. The quality of life index at the beginning was 50.7, increasing to 61.3 at six months of follow-up (*p* < 0.001; Cohen’s d = 0.84).

### 3.4. Costs of the Results Obtained

All costs associated with a patient having been given an ostomy in our autonomous community are gathered in Table 5. The cost per patient was €17,731, including the hospital stay and surgery, the follow-up treatment (chemotherapy and/or radiotherapy), costs of complications with the stoma (emergency services, specialist doctor and primary health care), and the care by APN-O. Moreover, indirect costs due to medical leave were included.

The therapeutic management of the base disease represents the top cost (46.9%), followed by hospital stay and surgery (41.9%). The indirect cost (medical leave) was 9.6% of the total, whereas complications with the stoma amounted for 0.8% of the total, and the cost of the care by APN-O was 0.77%.

Before the intervention with the nurses specialized in ostomy, the cost per patient was €17,450 (hospital stay, treatment and sick leave). The direct cost resulting from the new model increased by €281 (cost of complications with the ostomy-emergency room and hospital specialist visits- and follow-up by the APN-O).

### 3.5. Cost–Utility Analysis

To calculate the cost and utility, the difference in costs of following up an ostomy patient using the new model (inclusion of APN-O) or doing it exclusively from Primary Care, as in the previous clinical management model, has been taken into account.

Thus, the cost difference between both models was €136.99 and the utility gained was 0.05965. The efficiency (incremental cost–utility ratio) of ostomy patient care with the new clinical management model was €2297 per QALY gained.

### 3.6. Willingness to Pay and Cost–Benefit

The mean of WTP for a consultation by an ostomy nurse at the beginning of the study was €61 (range €0–170). At the end of the study, the mean of WTP was €69 (range €0–200). Despite the increase of more than 12%, there were no statistically significant differences (*p* = 0.142). However, differences were found between WTP and type of hospital (*p* = 0.014) and there were no differences between WTP and sex, previous work situation, or household income level. At the beginning of the study, 7.0% of the patients stated that they were not willing to pay. At the end of the study, the percentage of patients who stated that they were not willing to pay was 4.7%.

The official cost of each visit to an ostomy clinic for follow-up and management of complications is 21 euros (Table 1). Table 6 shows the WTP for the follow-up and control of the ostomy by the patients, finding statistically significant differences at the beginning and end (*p* < 0.001) and clinically relevant (Cohen’s d = 0.994).

If we take into account the mean cost of follow-up and stoma complications at current prices (€136.99/patient) and the cost that the patient is willing to assume for the same benefit obtained (€456.87/patient), the benefit/cost ratio would be 3.34.

## 4. Discussion

Attaining “value” for patients must become the general goal of healthcare provision. This value is defined as the health results obtained for each monetary unit spent [36]. If value improves, all parties involved may benefit (patients, payers, providers) whilst also increasing the health system’s sustainability.

Value must always be defined with the customer in mind and, in a health system that functions properly, value creation for patients must determine the rewards for all the system’s other stakeholders. Given that value depends on results, not on consumables, value in health care is measured by the results obtained, not by the volume of services provided. Therefore, the shift in focus from volume to value is a key challenge in current health systems.

### 4.1. Contributions in Terms of Value

Value is also not measured by the care process used. Measuring and improving the process are important tactics, but it does not substitute the measuring of results and costs. Thus, QALYs gained (value) since the start of our study were 0.05965, which amounts to a patient gaining more than 21 days (in six months) of good health (according to the QALY definition, best imaginable state of health). We found significant and (statistically) relevant differences from a clinical perspective both in gained utility (QALYs) and in quality of life measured with the specific questionnaire for ostomy patients (Montreux).

These results match those from other studies that reported significant improvements in HRQL when patients received care from a nurse specialized in ostomies after surgery [37,38,39]. The difference with other studies mainly revolves around the fact that our work values a new management process, not just patient interventions themselves. Utilities reflect patient preferences for health states and provide an alternate method of quality of life assessment for patients with stoma, although few studies have applied this method [40].

The percentage of patients with complications arising from the stoma (28.8%) suggests good management of surgical techniques and good health education in self-care. This is due to nurses specializing in ostomy care dedicated to training in self-care [13,41].

Our findings also match other studies that reported a more positive adjustment to a stoma after hospital discharge when patients received care from a nurse specialized in ostomies [12,13]. However, our study included a longer evaluation period for results than some of the mentioned studies (which only evaluated three months). This way, we further ensure the external validation of the results obtained. Therefore, focusing on outcomes (value-based healthcare) can help providers (Spanish National Health System) to reduce costs, provide better healthcare, and therefore increase efficiency, and all with limited resources.

### 4.2. Contributions in Terms of Cost (Reduction)

The cost, the equation denominator, refers to the total cost of the full cycle of care for the patient’s medical condition, not to the cost of individual services. The best focus for reducing cost usually involves spending more on some services in order to reduce the need for others.

Cost reduction without considering the results obtained is dangerous and self-destructive, leads to false “savings”, and potentially limits the efficacy of care.

Our results show that with this new management model, patients show better self-management, less adverse events, and a better evolution of HRQL. Moreover, patients showed a significant increase in their demand for consultations with specialized nurses and a decrease in demand for other more congested and specialized services, such as emergency hospital treatment, primary care, or hospital specialists. This supposes an important saving to the health system, in terms of both cost and time. Therefore, this new system of organization is more efficient than the previous one, it is profitable and greatly beneficial to patients.

The implementation of the new management model for patients with ostomies in our region offers a very efficient alternative: €2297/QALY. This is especially true if we consider that, in Spain, a technology, a new treatment or procedure, is considered to be efficient when the cost of gaining 1 QALY ranges between €22,000 and €24,000 [42].

In a “classic” scenario, before the implementation of the new management model, the patient is not followed by any specialized agent of the health system. Instead, they are referred up from the first level of health care (Primary Care) according to their complications. This brings about differences both in results and in costs. In terms of cost, the new model would show a decrease, even negative values, as opposed to the traditional one because the patient would have to be assisted by hospital care (hospital specialists) and by primary care (nurses and doctors). Thus, the model of organizational innovation based on APN-O has become an efficient alternative in a conservative scenario, which does not include the likely increase in costs associated with postoperative hospital stays, stoma complications, primary care visits, and emergency room visits among the control group patients [41].

### 4.3. Contributions in Terms of Economic Benefit

Patients’ WTP for care provided by nurses specialized in ostomies was €69 per consultation. If we consider the official prices of Andalusia, a patient is willing to pay up to three times the cost of consultations (ranging between 2.7 and 3.7 times the official price of hospital nursing consultations).

The percentage of patients who stated that they were not willing to pay for APN-O decreased at six months and the majority of them said they would be willing to pay for it. Although patients’ satisfaction was not analyzed, APN-O care it appears to be acceptable to patients. This fact confirms the high benefit obtained in our region with this new patient management model.

### 4.4. Limitations of Study

The main limitation of our study was that the economic evaluation was done without a control group. To overcome this limitation, we considered as a control group the cost and QALY obtained by the intervention group, considering the following assumptions: for the calculation of the cost, we considered the same consumption of medical resources except those derived from the new organizational model (not including APN-O). For the calculation of the QALY, we considered the baseline utility level, assuming that the patient’s quality of life remained constant throughout the study.

Other limitations have been possible, but we have tried to avoid them by carrying out the study prospectively, thus avoiding the memory bias, and by using a comprehensive data collection protocol that allowed us to collect all the main variables, the result of which is that there was hardly any important data missing.

With regard to the time horizon, six months, although it can be considered short, reality has shown us that it is enough time to highlight the changes related to the improvement of health outcomes.

Finally, another possible limitation would be the export of the management model to other regions of our country. However, we believe that this should not be a handicap, since all the regional health systems in our country operate in a very similar way, and even the costs do not differ in a high percentage. The model that has been explained briefly in methodology is easily assumed by any regional health system in our country, and could even be improved in other countries.

## 5. Conclusions

For clinical practice and management, it seems clear from the results of this study that the health authorities must be increasingly involved in evaluating what is the real support not only for interventions, but also for a new management model.

Our results show that an organizational innovation model based on the APN-O contributes to increasing value-based healthcare for ostomy patients, both because it proved to be effective in improving the health status of patients, and because of the low investment required by the system, which makes it highly efficient for the national health system of our country, especially if we also take into account that the willingness to pay for these services triples the investment of the health system.

For future research, this study can be used for benchmarking, not with the search for a ranking, but with the desire to improve by copying the best.

## Figures and Tables

**Table 1:** Costs used in the analysis.

Cost Classification	Costs in €	Reference
Day of hospital stay (general and digestive surgery)	603.70	25
Day of medical leave	24.80	27
Radiation therapy session	1087.34	25
IV chemotherapy session (2 h)	3924.61	25
IV chemotherapy session (2 h)	1438.00	28
Hospital emergency	144.24	25
Day of admission into the emergency ward	392.03	25
First hospital specialist consultation	114.12	25
Second and follow-up hospital specialist consultations	54.12	25
Hospital nursing consultation	20.69	26
Primary health care medical consultation	53.75	25
Primary health care nursing consultation	24.69	25
Primary health care emergency	83.65	25
**SMW: spanish minimum wage**	**735,9/month**	29

**Table 2:** Patients sociodemographic characteristics.

Variable	Characteristics	Total (n = 75)	Men (n = 42)	Women (n = 33)	Value of *p*
Age in years (range)		61 (25–90)	62 (25–90)	59 (28–87)	0.389
Civil status (%)	Single	18.7%	50.0	50.0	0.802
	Married (with partner)	70.7%	58.5	41.5	
	Separated or divorced	4.0%	66.7	33.3	
	Widowed	6.7%	40.0	60.0	
Hospital type (%)	County	24.0%	55.6	44.4	0.911
	Specialized	62.7%	57.4	42.6	
	Regional	13.3%	50.0	50.0	
Level of education (%)	No education	20.6%	42.9	57.1	0.508
	Primary	35.3%	62.5	37.5	
	Secondary	30.9%	47.6	52.4	
	University	13.3%	66.7	33.3	
Employment regime (%)	Housewife	33.3%	0.0	100	<0.001
	Employed	53.3%	65.6	34.4	
	Self-employed	11.7%	100	0.0	
	Student	1.7%	0.0	3.1	
Previous employment situation (%)	Active	12.0%	66.7	33.7	<0.001
	Unemployed	12.0%	66.7	33.7	
	Medical leave	13.3%	60.0	40.0	
	Home care	14.7%	0.0	100	
	Retiree/pensioner	42.7%	75.0	25.0	
Income level (%)	<1 SMW	37.3%	32.1	67.9	0.006
	1–2 SMW	37.3%	71.4	28.6	
	>2 SMW	25.3%	68.4	31.6	

**Table 3:** Patients clinical characteristics.

Variable	Characteristics	Total	Men % (n = 42)	Women % (n = 33)	Statistical Significance
Reason for the surgery	Oncological	74.7%	55.4	44.6	0.837
	Inflammatory bowel disease	17.3%	53.8	46.2	
	Familial polyposis	1.3%	100	0.0	
	Other	6.7%	60.0	40.0	
Type of surgical intervention	Programmed	59.5%	56.8	43.2	0.476
	Urgent	40.5%	53.3	46.7	
Type of ostomy	Colostomy	59.4%	58.5	41.5	0.584
	Ileostomy	39.1%	51.9	48.1	
	Urostomy	1.4%	100	0	
Postoperative treatment	No treatment	38.8%	53.8	46.2	0.508
	Radiation therapy	4.5%	33.3	66.7	
	Oral chemotherapy	13.4%	44.4	55.6	
	IV chemotherapy	11.9%	50.0	50.0	

**Table 4:** Ostomy complications by hospital type.

Hospital Type	Patients with Complications		Value of *p*
	YES	NO	
County	82.4%	17.6%	0.044
Specialized	59.0%	41.0%	
Regional	33.3%	66.7%	

**Table 5:** Costs of an ostomized patient.

	Cost (€)	n per Patient	% of Patients	Cost per Patient
**Hospital care**				**15,752.35**
Hospital stay (days)	603.70	12.29	100	7421.34
Treatment cost				8331.01
IV chemotherapy	3924.61	5.76	25.76	5827.99
oral chemotherapy	1438.00	5.10	24.24	1777.71
radiation therapy	1087.34	8.80	7.58	725.30
**Cost of complications and follow-up**				281.06
**Hospital care**				**102.22**
emergency hospital treatment	144.24	1.43	10.60	21.84
admission into emergency ward observation	392.03	2.00	3.03	23.76
first specialized care visit	114.12	1.00	30.30	34.58
second and further visits	54.12	2.24	18.18	22.04
**Primary health care**				**41.85**
doctor	53.75	4.20	7.58	17.10
nurse	24.69	4.88	12.12	14.60
emergency ward	83.65	1.60	7.58	10.14
**APN-ostomy follow-up**				**136.99**
planned consultations	20.69	4.82	98.48	98.24
telephone consultations		2.77	54.55	31.23
consultations upon request (no prior appointment)		1.42	18.18	5.33
emergency consultations		1.40	7.58	2.19
**Direct medical costs**				16,033.41
**Indirect costs** (sick leave, days)	24.80	251	27.27	1697.67
**TOTAL COSTS**				**17,731.08**

**Table 6:** Willingness to pay (WTP) for each ostomy-specialized advanced practice nurse (APN-O) follow-up consultation and consultation for complications control in patients with ostomies.

	WTP €0	From €1 to 25	From €25 to 50	From €50 to 75	From €75 to 100	More than €100	Value of *p* (chi-squared)
	% of patients						
**Start**	7.0	7.0	42.1	14.0	21.1	8.8	<0.001
**End**	4.5	-	43.9	21.2	19.7	10.6	
